# The characterization of *Thermotoga maritima* Arginine Binding Protein variants demonstrates that minimal local strains have an important impact on protein stability

**DOI:** 10.1038/s41598-019-43157-y

**Published:** 2019-04-29

**Authors:** Nicole Balasco, Giovanni Smaldone, Marilisa Vigorita, Pompea Del Vecchio, Giuseppe Graziano, Alessia Ruggiero, Luigi Vitagliano

**Affiliations:** 1 Institute of Biostructures and Bioimaging, CNR, Via Mezzocannone 16, Napoli, Italy; 2 IRCCS SDN, Via Emanuele Gianturco 113, Napoli, Italy; 3 Department of Science and Technology, University of Sannio, via Port’Arsa 11, Benevento, Italy; 4 Department of Chemical Sciences, University of Naples Federico II, via Cintia, Napoli, Italy

**Keywords:** X-ray crystallography, Biophysics

## Abstract

The Ramachandran plot is a versatile and valuable tool that provides fundamental information for protein structure determination, prediction, and validation. The structural/thermodynamic effects produced by forcing a residue to adopt a conformation predicted to be forbidden were here explored using *Thermotoga maritima* Arginine Binding Protein (TmArgBP) as model. Specifically, we mutated TmArgBP Gly52 that assumes a conformation believed to be strictly disallowed for non-Gly residues. Surprisingly, the crystallographic characterization of Gly52Ala TmArgBP indicates that the structural context forces the residue to adopt a non-canonical conformation never observed in any of the high-medium resolution PDB structures. Interestingly, the inspection of this high resolution structure demonstrates that only minor alterations occur. Nevertheless, experiments indicate that Gly52 replacements in TmArgBP produce destabilizations comparable to those observed upon protein truncation or dissection in domains. Notably, we show that force-fields commonly used in computational biology do not reproduce this non-canonical state. Using TmArgBP as model system we here demonstrate that the structural context may force residues to adopt conformations believed to be strictly forbidden and that barely detectable alterations produce major destabilizations. Present findings highlight the role of subtle strains in governing protein stability. A full understanding of these phenomena is essential for an exhaustive comprehension of the factors regulating protein structures.

## Introduction

Protein structures are endowed with a remarkable conformational versatility. Even for proteins of limited size, the number of possible conformations is so huge that it is very difficult to understand how the polypeptide chain can find the final folded state through exhaustive exploration of the conformational space (Levinthal paradox)^1–4^. The conformational versatility of the protein backbone is generated by the possibility to modulate the dihedral angles φ, ψ, and ω that display a diversified level of variability. The values accessible to the ω angle, which describes the planarity of the peptide bond, are generally restricted to either 180° or, more rarely, to 0°. The discovery of this property has been crucial for the prediction of basic features of protein secondary structure elements. In principle, the two angles (φ, ψ) should not have strong restrictions, as they describe rotations along single bonds. However, the seminal work by the Ramachandran group^5–7^, corroborated by independent analyses by Liquori and cowokers^8^, demonstrated that specific combinations of (φ, ψ) are forbidden due to severe steric clashes between atoms of the protein chain. The elaboration of the so-called Ramachandran plot, that reports allowed and forbidden regions in the (φ, ψ) space, has been one of the major achievements in structural biology^9^. Even though the establishment of limitations on the values accessible to main chain angles did not solve the Levinthal paradox, it had an enormous impact in protein structure determination, modelling, and validation. Over the years, many efforts have been devoted to the definition of boundaries between allowed and forbidden regions in the Ramachandran plot^10–17^. Although different studies have reached (slightly) different conclusions, there is a consensus about the concept that some regions of the Ramachandran plot are strictly forbidden. According to Brasseur and coworkers^10^, strictly forbidden regions correspond to the (φ, ψ) combinations that generate multiple steric clashes within the polypeptide chain. Using this insightful concept, they were able to define reliable boundaries for the allowed regions of the plot. Regions forbidden to all types of residues are very frequently associated with (φ, ψ) values in which one or both dihedrals are close to 0°. For non-Gly residues the presence of the C^β^ generates other potential clashes that create strictly forbidden regions also for conformations in which both φ and ψ are very different from zero. This region is located in the lower right quadrant of the Ramachandran plot, with dihedral angles falling in the intervals: 90°/180° and −180°/−80° and includes the so-called ε region that is populated only by Gly residues^10,18^. In this ε region, the local conformation generates clashes of the C^β^ atom with both O_i−1_ and H_i+1_.

**Figure 1:**
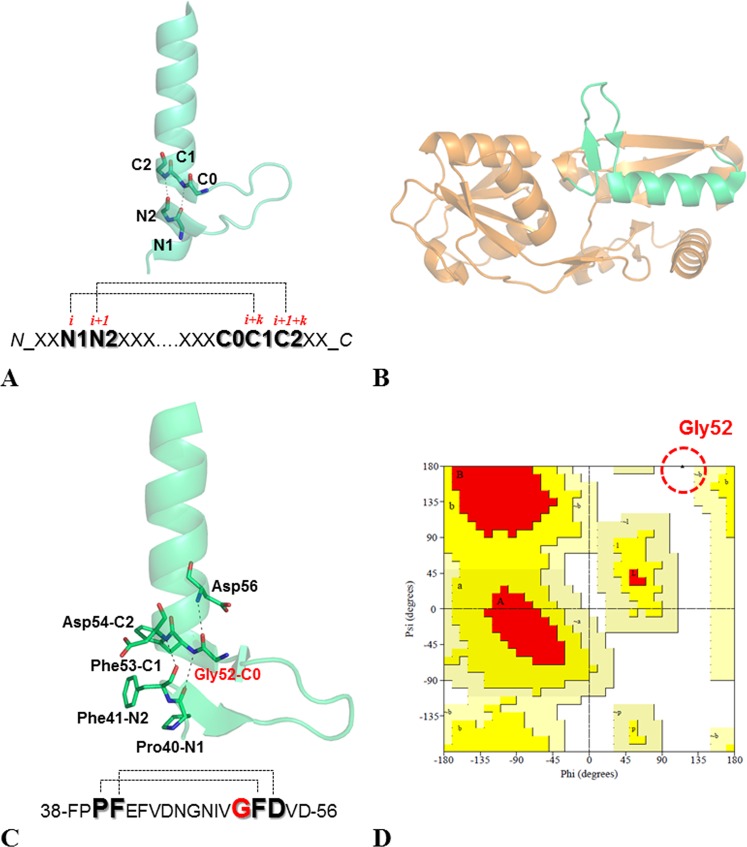
The pattern of the H-bonds that lock the helix insertion motif within a cartoon representation of a representative example (PDB ID: 3AYF) are reported in panel A. Location of the insertion motif (in green) in the crystal structure of the arginine-bound TmArgBP^20-233^ (orange, PDB ID: 6GGV) (**B**). Snapshot of the local structure of the insertion within the α-helix in TmArgBP^20-233^ (**C**). The protein sequence highlighting the residues involved in the H-bonds and the location of the Glycine residue in C0 (in red) are reported. Ramachandran plot showing the conformation adopted by Gly52 in TmArgBP^20-233^ (PDB ID: 6GGV) computed using Procheck (**D**)^31^.

**Figure 2:**
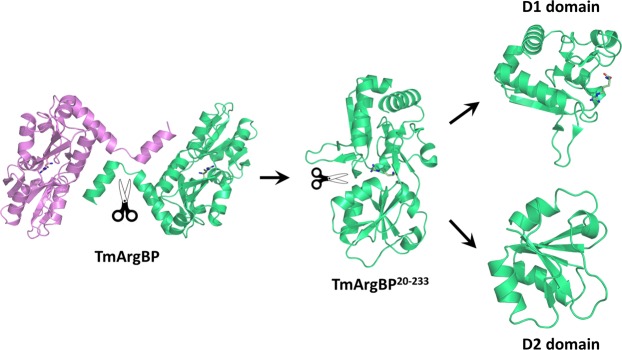
Schematic cartoon representation of the protein dissection of the arginine-bound TmArgBP protein: TmArgBP swapping dimer (PB ID: 4PSH), TmArgBP^20-233^ truncated monomer (PB ID: 6GGV), D1 (PB ID: 6GPC) and D2 (PB ID: 6GPM) individual domains. The arginine ligand is shown in sticks.

In the search for novel protein structural states, we recently detected and characterized a novel motif consisting in the insertion of fragments within α-helices (Fig. 1A)^19^. These insertions displayed a large variety of size, sequence and structure. We also discovered that the formation of this structure is favored by the presence of a Gly that precedes the residues that lock the motif by forming H-bonds (Fig. 1A). Many of these Gly residues adopt conformations that are forbidden to non-Gly residues, and frequently fall in the ε region^19^. Among others, the arginine binding protein isolated from *Thermotoga maritima* (TmArgBP) contains one helix insertion motif (Fig. 1B–C), with Gly52 in the C0 position. It is worth noting that, in all the TmArgBP variants characterized so far by X-ray cristallography^20–22^, Gly52 residues adopt a conformation forbidden to non-Gly residues, with (φ, ψ) ∼ (130°, ±180°) (Figs 1D and S1). It has been shown that the remarkable thermal and chemical stability of TmArgBP allows major manipulations without disrupting its folded state (Fig. 2)^20–23^. In this framework, we decided to use TmArgBP as a model system to investigate the effect of replacing Gly52 with non-Gly residues. In principle, these mutations could generate two alternative scenarios characterized either (a) by a deformation of the protein local structure with a shift of the non-Gly residue in the allowed region of the Ramachandran plot, or (b) by preservation of the local structure with non-Gly residues falling in the forbidden region of the Ramachandran plot. Present data demonstrate that this strictly forbidden conformation is indeed accessible to Ala52 in the specific structural context of TmArgBP. A close inspection of the mutation site indicates that

protein destabilization may be achieved with minimal structural perturbations.

## Results and Discussion

### Design and expression of TmArgBP Gly52 mutants

The structural effects associated with the replacements of Gly residues adopting conformations forbidden to other residues were here evaluated using TmArgBP as model system. This protein is endowed with remarkable biophysical and structural properties^20–22^, and presents a helix insertion motif located in the D1 domain (Fig. 1B–C). The helix insertion motif is locked by two consecutive C = O H-N H-bonds, made by residues Pro40-Phe53 and Phe41-Asp54. The inserted region has a β-hairpin conformation and consists of 10 residues. The Gly52 residue, which occupies the C0 position of the motif and presents a well-defined electron density in the crystal structures of TmArgBP variants, adopts a conformation that is strictly forbidden to non-Gly residues (Fig. 1D and S1). Gly52 is also involved in a main chain-main chain H-bond with the helix residue Asp56; this H-bond likely stabilizes the protein local structure. As shown in Fig. 2, wild-type TmArgBP exists as a dimer due to domain swapping of the C-terminal α-helix. It also forms oligomers by means of domain swapping. Thus, to avoid protein heterogeneity, the mutagenesis studies have been conducted on the truncated and consequently monomeric form of TmArgBP (residues 20–233, TmArgBP^20-233^) (Fig. 2). The Gly52 residue in C0 position was replaced either by Ala (TmArgBP^20-233_G52A^) or by Val (TmArgBP^20-233_G52V^). TmArgBP^20-233_G52A^ was designed to explore the effect of a minimal perturbation of the site, whereas a larger destabilization was expected for TmArgBP^20-233_G52V^ because Val has a bulkier side chain and a slightly more restricted conformational space compared to Ala.

Both recombinant proteins were expressed in *E. coli* cells, and were purified to homogeneity in amounts that allowed structural and biophysical investigations. For TmArgBP^20-233_G52A^, in addition to the arginine-bound form that is usually obtained from the purification procedure^20,21^, the ligand-free form was also prepared by adopting the devised protocol^20,24^.

### CD characterization of TmArgBP^20-233_^^G52A^ and TmArgBP^20-233_^^G52V^

**Figure 3:**
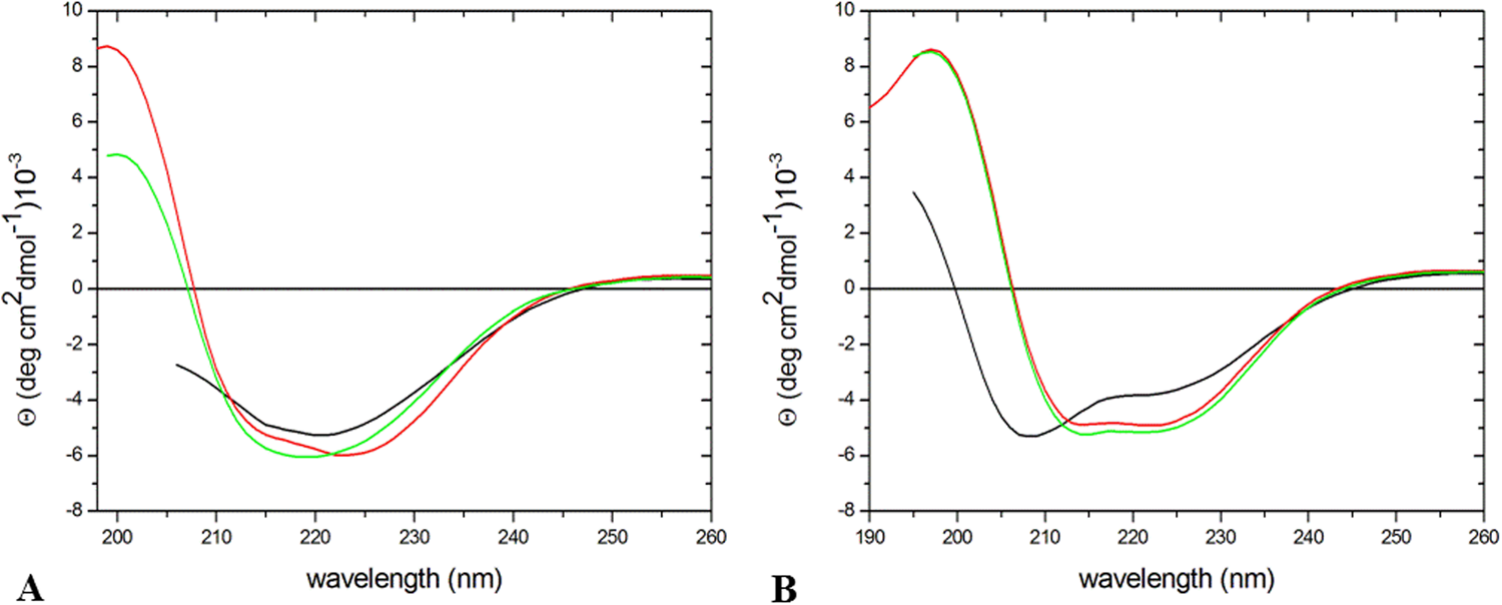
Far-UV CD spectra of the arginine-bound forms of TmArgBP^20-233_G52A^ (**A**) and TmArgBP^20-233_G52V^ (**B**) in PBS buffer at 20 °C (red line), at 100 °C (black line) and after thermal denaturation at 20 °C (green line).

**Figure 4:**
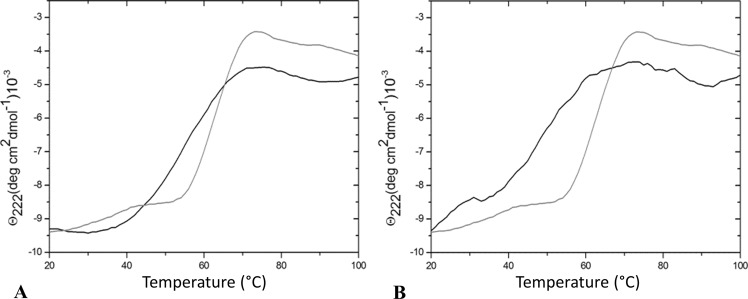
Effect of the Gly52Ala and Gly52Val mutations on the thermal denaturation of TmArgBP^20-233^. The change in ellipticity [θ] at 222 nm when the temperature increases from 20 °C to 100 °C was monitored for TmArgBP^20-233_G52A^ (**A**) and TmArgBP^20-233_G52V^ (**B**) in the ligand bound state (black line). The denaturation profile of the parent protein TmArgBP^20-233^ is reported in grey. The samples were dissolved in PBS buffer pH 7.4 containing 4 M GuHCl.

The folded state of the two mutants was initially investigated by circular dichroism (CD) spectroscopy. Inspection of the far-UV CD spectra indicates that the two variants retain the α/β structure of the parent protein (Fig. 3A,B). To shed light on the effects of mutation on the conformational stability of the protein, far-UV CD spectra were collected at high temperature. Spectra collected at 100 °C showed limited variations compared to those collected at 20 °C; cooling of these samples to room temperature essentially restores the initial spectral features. These observations are confirmed by the analysis of temperature-induced denaturation curves, obtained by recording the molar ellipticity at 222 nm, that do not present any indication of a conformational transition over the 20–100 °C temperature range (Fig. S2A,B). These data demonstrate that both variants are highly thermostable proteins, despite the Gly52 replacement. In line with the parent protein^21^, significant alterations of the molar ellipticity at 222 nm could be obtained by increasing the temperature in the presence of GuHCl. A temperature-induced conformational transition is observed when the two mutants are heated in the presence of 4 M GuHCl (Fig. 4A,B). The denaturation curve of the arginine-bound form of TmArgBP^20-233_G52A^ suggests that the protein undergoes a rather cooperative denaturation, although less cooperative than that of the parent protein TmArgBP^20-233^, with a mid-point temperature of 54 °C (Fig. 4A). The comparison of the mid-point temperature of this variant with that exhibited by the parent TmArgBP^20-233^ indicates that a destabilization of about 12 °C is produced by the Gly52Ala mutation (Table 1). As observed for the parent TmArgBP^20-233^ protein^21^, the ligand-free form of TmArgBP^20-233_G52A^ is more stable than the arginine-bound one (63 °C *versus* 54 °C). The characterization of the arginine-bound form of TmArgBP^20-233_G52V^ in the same experimental conditions highlights some significant differences. The temperature-induced variation of the CD signal of this form is less cooperative than that of TmArgBP^20-233_G52A^, and the Gly52Val mutation causes a larger destabilization (i.e., the mid-point temperature is 49 °C) (Fig. 4B).

**Table 1:** Mid-point temperatures (T_d_) of TmArgBP^20-233^, TmArgBP^20-233_G52A^, and TmArgBP^20-233_G52V^ obtained from the far-UV CD measurements at pH 7.4 in the presence of 4 M GuHCl.

	T_d_ (°C)	
	Arginine-bound	Ligand-free
TmArgBP^20-233^	66	72
TmArgBP^20-233_^^G52A^	54	63
TmArgBP^20-233_^^G52V^	49	—

### DSC characterization of TmArgBP^20-233_G52A^ and TmArgBP^20-233_G52V^

**Figure 5:**
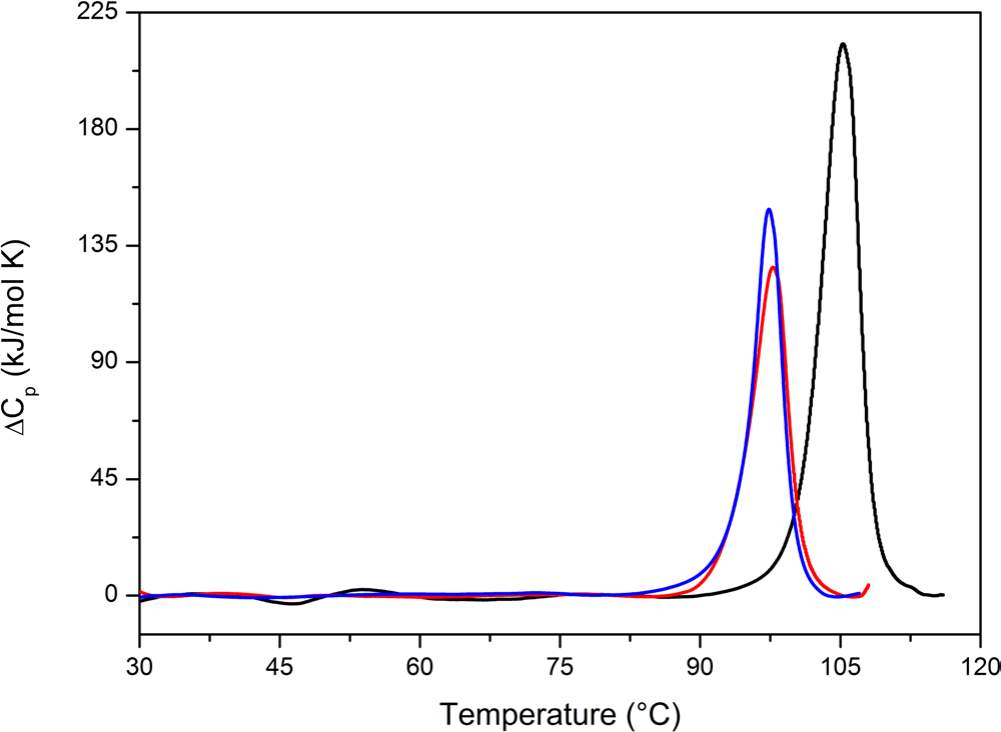
DSC measurements of TmArgBP^20-233^ (black line), TmArgBP^20-233_G52A^ (red line) and TmArgBP^20-233_G52V^ (blue line) in the ligand bound state. All samples were diluted in PBS buffer at pH 7.4.

To enlarge the temperature range explored by far-UV CD measurements, and to characterize the conformational stability of the two variants in the absence of GuHCl, differential scanning calorimetry (DSC) measurements were performed on the Gly52Ala and Gly52Val mutants of TmArgBP^20-233^. It was already shown that the thermal denaturation of the parent protein TmArgBP^20-233^ is irreversible when the sample is heated up to 120 °C^21^. However, by stopping the first heating at the T_d_ value, the second heating is characterized by a DSC peak whose area amounts to 75% of that of a single complete scan. When similar experiments were performed on the arginine-bound form of TmArgBP^20-233_G52A^, a DSC peak, whose area was about to 60% of that of a single complete scan, was obtained (data not shown). This observation indicates that the irreversibility is due to the high temperatures at which the samples elapse for many minutes^25–27^. This allows the treatment of the temperature-induced denaturation of these species as a reversible process. Table 2 reports the values of the denaturation temperature, the denaturation enthalpy change, the van’t Hoff enthalpy change and their ratio (CU) for TmArgPB^20-233^ and its mutants in the ligand bound state. The process can be considered reversible and well described by the two-state N ↔ D transition model because the CU value is close to one, a necessary condition for the occurrence of a two-state transition^28,29^. Comparison between the DSC profiles of TmArgBP^20-233_G52A^ and TmArgBP^20-233_G52V^ with that of the parent protein in their ligand bound state indicates that the destabilization induced by the single mutation is remarkable (Fig. 5). The T_d_ values of both mutants are less than 98 °C to be compared to 105 °C of TmArgBP^20-233^ and there is also a Δ_d_H(T_d_) decrease of about 350 kJ/mol (Table 2). Even though the T_d_ values seem to suggest that there is no stability difference between the two mutants, it should be noted that TmArgBP^20-233_G52V^ is more prone to aggregation as the sample after thermal denaturation appears cloudy. This observation is corroborated by the characterization of a TmArgBP^20-233_G52V^ sample stored at 4 °C for 2 days. The DSC trace shows an abrupt change at about 45 °C where a non-cooperative destabilization occurs; this event is not present in fresh samples of TmArgBP^20-233_G52V^ (Fig. S3).

**Table 2:** Experimental values of denaturation temperature Td, denaturation enthalpy change ΔdH(Td) and cooperative unit CU = ΔH(T_d_)/ΔH(T_d_)^vH^ obtained for TmArgBP^20-233^, TmArgBP^20-233_G52A^, and TmArgBP^20-233_G52V^ from the DSC analysis.

	T_d_ (°C)	Δ_d_H(T_d_) (kJ mol^−1^)	CU	Δ_d_H^vH^ (kJ mol^−1^)
TmArgBP^20-233^	105.3	1000	1.0	995
TmArgBP^20-233_G52A^	97.8	652	0.97	674
TmArgBP^20-233_G52V^	97.7	648	0.87	765

DSC data indicate that the Gly52Ala mutation induces a decrease in protein stability in line with far-UV CD measurements in the presence of 4 M GuHCl. The decrease in Δ_d_H(T_d_) could be due to the unfavorable interactions of Ala side chain with adjacent residues (see below). The Gly52Val mutation does not have additional effects on T_d_ or Δ_d_H(T_d_), but it renders the protein more prone to aggregate. This effect, which is in line with the non-cooperative unfolding observed in far-UV CD experiments conducted in the presence of 4 M GuHCl, is likely due to a greater local destabilization induced by the large Val side chain. These findings demonstrate that the replacement of Gly52 has a remarkable impact on the conformational stability of TmArgBP^20-233^.

### Crystal structure of TmArgBP^20-233_G52A^

To unravel the impact of the Gly52 mutations on TmArgBP^20-233^ structure, crystallization experiments were undertaken on both mutants. Crystals suitable for X-ray diffraction analyses were grown for the arginine-bound form of TmArgBP^20-233_G52A^ using ammonium sulfate as precipitating agent (see Methods for details). Despite the extensive attempts made, we were not able to grow crystals for arginine-bound TmArgBP^20-233_G52V^. This result should be considered a clear indication of the large local destabilization induced by the Gly52Val mutation.

**Figure 6:**
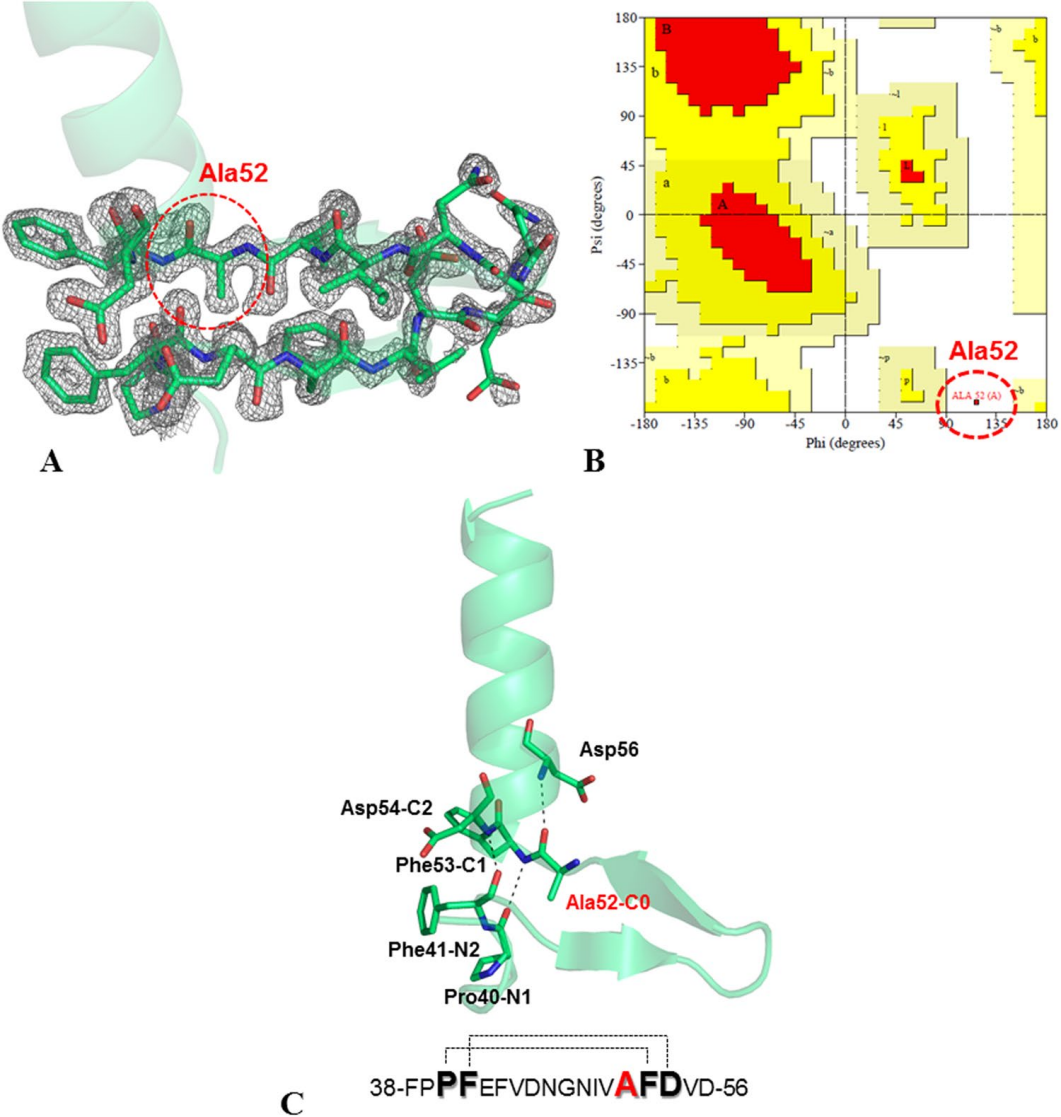
|2Fo-Fc| omit map, contoured at 1.5σ, of the region carrying the mutation Gly52Ala in the crystal structure of the arginine-bound form of TmArgBP^20-233_G52A^ (**A**). Ramachandran plot showing the conformation adopted by Ala52 in TmArgBP^20-233_^^G52A^ computed using Procheck (**B**)^31^. Cartoon representation of the helix insertion present in the structure of the mutant TmArgBP^20-233_G52A^ (**C**). The residues (Pro40, Phe41 and Phe53, Asp54) making the consecutive pair of (CO NH) H-bonds are represented. The Ala52 residue in C0 is also shown.

High resolution data, extending up to 1.64 Å resolution, were collected (Table 3) on TmArgBP^20-233_G52A^ crystals. The refined model displays good values for the crystallographic indicators (Table 4). The TmArgBP^20-233_G52A^ crystals contain one molecule in the asymmetric unit and, as expected, the protein presents an arginine molecule in the active site (Fig. S4). The overall and local structure of the protein is not affected by the single mutation. Indeed, the root mean square deviation (RMSD) value computed on the C^α^ atoms between TmArgBP^20-233_G52A^ and TmArgBP^20-233^ is as low as 0.29 Å, although the crystals used to solve these structures were not isomorphous. Inspection of the electron density maps in the mutation site clearly shows the presence of an Ala residue in position 52. As shown in Fig. 6A, the entire helix insertion region presents a well-defined electron density. Determination of Ala52 main chain dihedral angle values (φ = 117.3°, ψ = −170.9°) indicates that Ala52 assumes a conformation closely similar to that of Gly52 in TmArgBP structures (Table S1 and Fig. S1). This conformation falls in the ε region of the Ramachandran plot, and is considered to be strictly forbidden to non-Gly residues (Fig. 6B). In line with this consideration, commonly used validations protocols, such as Molprobity^30^ and Procheck^31^, denote Ala52 as a structural outlier since it displays very unusual (φ, ψ) angles for non-Gly residues. This conclusion is also corroborated by an analysis of the entire medium/high resolution structural content of the Protein Data Bank (release of November 2016). The occurrence analysis of conformations with (φ, ψ) dihedrals falling in a 10°×10°-box centered at (117°, −170°) for Gly and non-Gly residues in 45,214 crystal structures refined at a resolution better than 2.0 Å found: (a) 1,825 out of 1,526,122 Gly residues adopt a conformation falling in this box; (b) none of the 17,862,412 non-Gly residues adopts (φ, ψ) values falling in this box. In other words, this conformational state is never populated by non-Gly residues. It is worth noting that this region is not populated either in MD simulations carried out on the Ala dipeptide using different force fields^32^.

**Table 3:** Data collection statistics. Values in parentheses are for the highest resolution shell (1.70–1.64 Å).

	TmArgBP^20-233_G52A^
X-ray device	Rigaku FR007HF with CCD detector
Space group	C2
Unit cell parameters a, b, c (Å) β (°)	92.57, 52.24, 59.89 117.39
Resolution range (Å)	50.00–1.64
Wavelength (Å)	1.54
Average redundancy	5.1 (2.9)
Unique reflections	30030
Completeness (%)	96.1 (79.5)
R_merge_ (%)	6.4 (19.6)
Average I/σ(I)	30.3 (4.0)
Mosaicity (deg.)	0.51
R_meas_	0.070 (0.241)
R_pim_	0.028 (0.137)
CC* highest shell	0.985
CC_1/2_ highest shell	0.943

**Table 4:** Refinement statistics.

	TmArgBP^20-233_G52A^
Resolution range (Å)	15.00–1.64
Asymmetric unit	Monomer
R, R_free_ (%)	16.3, 18.9
No. of residues	213
No. of ligand molecules	1
No. of water molecules	272
Diffraction Precision Index (Å)	0.082
**Mean B values (Å** ^**2**^ **)**	
protein	14.6
ligand	8.2
water	27.0
**Mean occupancy factors**	
protein	0.987
ligand	1
water	0.998
**R.m.s. deviation**	
bond lengths (Å)	0.024
deviation bond angles (°)	2.234
**Isotropic Thermal Factor Restraints**	
main-chain bond refined atoms (Å^2^)	1.403
side-chain bond refined atoms (Å^2^)	2.743
Long range B refined atoms (Å^2^)	6.103
**Procheck**	
Ramachandran most favored	175 (91.6%)
Ramachandran additional allowed	15 (7.9%)
Ramachandran generously allowed	0 (0%)
Ramachandran disallowed	1 (0.5%)
**MolProbity**	
Ramachandran Favored	205 (97.2%)
Ramachandran Allowed	5 (2.4%)
Ramachandran Outliers	1 (0.4%)
Favored rotamers	181 (96.8%)
Allowed rotamers	3 (1.6%)
Outlier rotamers	3 (1.6%)

In this scenario, the local structure of the protein has deeply been scrutinized to identify the structural constraints that generated this unusual conformational behavior. As shown in Fig. 6C, the helix insertion motif is well preserved in the structure of the mutant. Indeed, residues Pro40-Phe53, and Phe41-Asp54 make the couple of consecutive C = O H-N H-bonds stabilizing this structural state. Moreover, as in the parent protein, the carbonyl-oxygen of Ala52 forms an H-bond with the nitrogen of Asp56.

**Figure 7:**
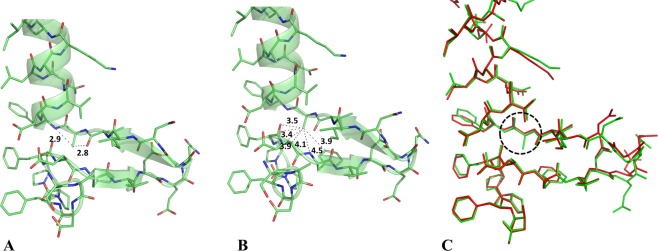
Distances in Å of the C^β^ atom of Ala52 with the carbonyl-oxygen of the previous residue Val51 and the nitrogen atom of the following residue Phe53 in TmArgBP^20-233_G52A^ (**A**). Distances between the C^β^ atom of Ala52 and surrounding residues in the structure of TmArgBP^20-233_G52A^ are reported (**B**). Superimposition of the helix insertion motif region in TmArgBP^20-233^ (red) and TmArgBP^20-233_G52A^ (green) (**C**).

As mentioned above, the ε region is forbidden to non-Gly residues for the unfavorable contacts of the C^β^ atom with the carbonyl-oxygen of the previous residue and the nitrogen atom of the following residue. The analysis of the present crystal structure does confirm the occurrence of close contacts between these groups. In TmArgBP^20-233_G52A^ the distances Ala52C^β^-Val51O and Ala52C^β^-Phe53N are 2.8 Å and 2.9 Å, respectively (Fig. 7A). Since it has been shown that backbone geometry may show a significant level of variability, the local valence bond geometry and deviations from planarity of the peptide bond have been compared with values detected in well refined protein structures (Table S1). This analysis unravels the occurrence of significant deviations from average values for both peptide planarity and bond angles. The deviation of the NC^α^C^β^ is particularly remarkable as it has never been observed in the structures (or occurs very rarely). This deformation is the local structure response to the strain caused by the close contacts described above. Moreover, in TmArgBP^20-233_G52A^ the C^β^ atom of Ala52 points toward the helix insertion. Fig. 7B,C indicate that a minimal rearrangement of the local structure allows the accommodation of Ala52 side chain without major steric clashes, as all the C^β^ atom distances with surrounding atoms are larger than 3.4 Å.

Altogether these findings demonstrate that the significant destabilization induced by the Gly52Ala mutation is caused by apparently minimal structural perturbations.

### MD simulations of TmArgBP^20-233^ and TmArgBP^20-233_G52A^

**Figure 8:**
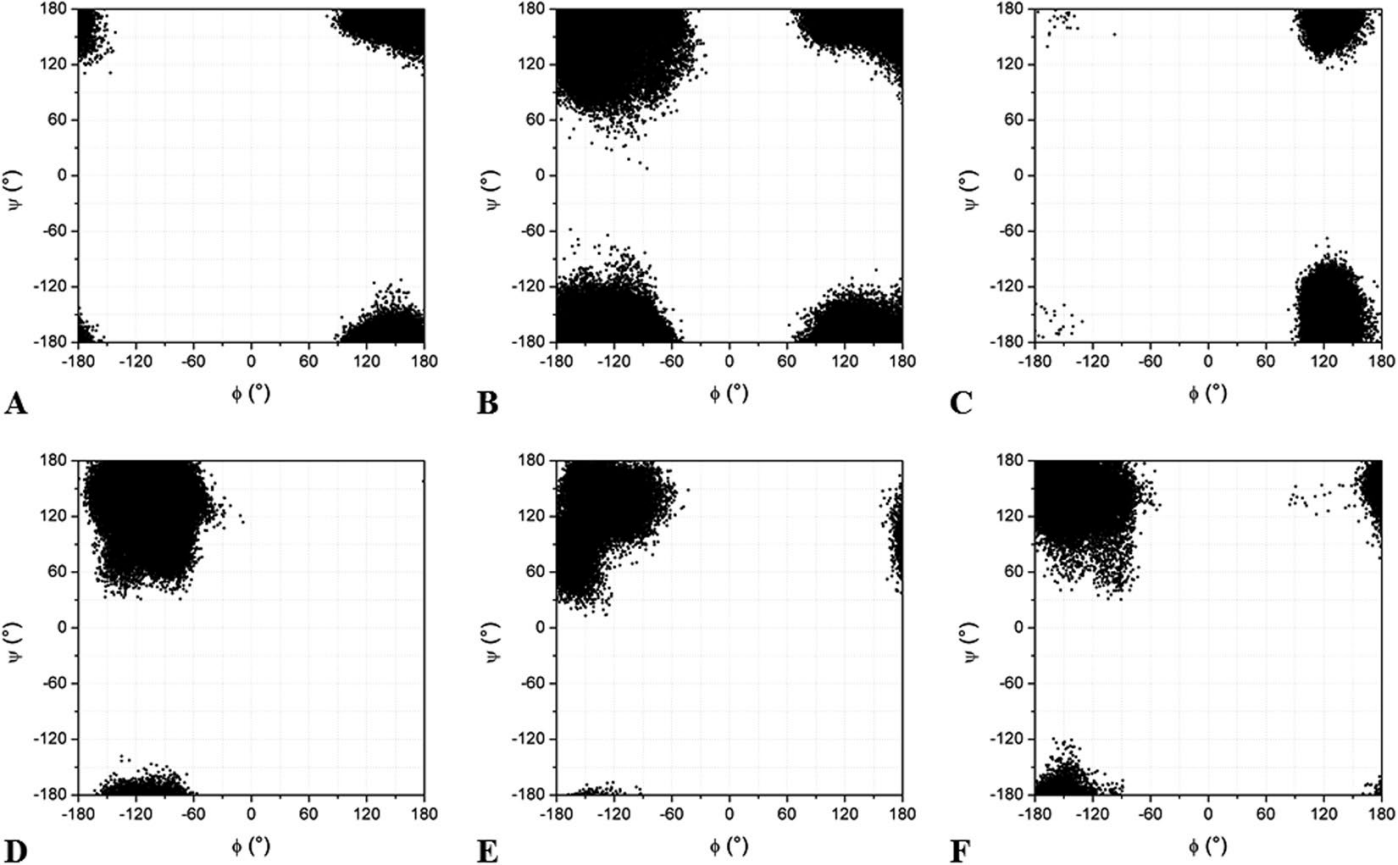
Ramachandran plots showing the conformations adopted by Gly52 (**A–C**) or Ala52 (**D–F**) in the trajectory structures obtained in the equilibrated region of MD trajectories (50-150 ns) in the simulations of TmArgBP^20-233^ and TmArgBP^20-233_G52A^ performed with three different force fields: AMBER99SB (**A,D**), CHARMM27 (**B,E**), OPLS-AA (**C,F**).

The crystal structure of TmArgBP^20-233_G52A^ shows that the Ala52 conformation is extremely unusual. To test the ability of tools commonly used in molecular modeling and dynamics to reproduce this peculiar state, MD simulations were performed on both the parent protein and the mutant by using three widely used protein force fields (namely, AMBER99SB^33^, CHARMM27^34^ and OPLS-AA^35^), and their associated optimized explicit water models (Table S2). Although, as mentioned above, MD simulations on the Ala dipeptide showed that this region was not populated^32^, present simulations, performed on the entire protein, were aimed at verifying whether the structural context was able to change this scenario. MD simulations, each 150 ns long, were performed using GROMACS software package^36^. All systems reached a stable state, as indicated by the time evolution of several parameters such as the RMSD against the starting model (Fig. S5A,C), the gyration radius (Fig. S5B,D), and the secondary structure (Figs S6 and S7). The analysis of the simulations conducted on TmArgBP^20-233^, in which a Gly is present in the C0 position, indicates that, in the three independent runs carried out using different force fields, this residue frequently adopts conformations falling in the ε-region both in the initial stages and in the equilibrated region of the trajectory (50–150 ns), in line with experimental structural data (Figs 8A–C and S8A–C). A different picture emerges from the inspection of the TmArgBP^20-233_G52A^ simulations. In this case, the simulation performed using the OPLS-AA force field displays rather large RMSD values in the equilibrated region (Fig. S5C). This is due to a significant alteration of the helix insertion motif. Indeed, for this simulation the β-structure of the insertion motif in TmArgBP^20-233_G52A^ was rapidly lost (Fig. S7C). On the other hand, the secondary structure of this motif is well preserved in trajectories obtained using AMBER99SB and CHARMM27 (Fig. S7A,B). The local flexibility of the trajectory structures are in line with those observed in the crystalline state (Fig. S9). We generated the standardized B-factors (see the definition of O. Carugo)^37,38^ from the equilibrated region of the trajectories that were compared with standardized B-factors derived from the crystal TmArgBP^20-233_G52A^ structure. The comparisons show a general agreement between the computed and the experimental trends.

The ability of these force fields to reproduce the conformation adopted by the residue in the C0 position of the helix insertion motif was specifically evaluated by analyzing the (φ, ψ) values of the residue in position 52 in the trajectory structures. In all cases, Ala52 conformations falling in the ε-region are detected only in the early stage of the MD simulations (Fig. S8D–F) as they occur in the pre-equilibration region of the trajectory and disappear in the equilibrated region (Fig. 8).

A deep inspection of the MD trajectories indicates that the occurrence of Ala52 in the ε-region is detectable only in the first 10 ns of the AMBER99SB and OPLS-AA simulations whereas it is lost in the first ns of the CHARMM27 simulation. Similar results are obtained in all the second independent MD runs performed on TmArgBP^20-233_G52A^ with the three different force-fields (Fig. S10). Also in these cases, the location of Ala52 in the forbidden region is lost within the first 7 ns. This finding indicates that the peculiar conformational state of Ala52 is progressively shifted toward more canonical regions of the Ramachandran plot and that widely used force fields are essentially unable to reproduce the structural state observed in the X-ray structure.

### Concluding remarks

A full understanding of the factors that drive backbone conformations in proteins is crucial in different areas of structural biology^39^. The impact of replacing Gly52, a residue adopting a conformation that is considered to be strictly forbidden to other residues, with Ala/Val has been investigated in the truncated monomer of TmArgBP protein. The characterization of the Ala mutant indicates that the structural context is able to force the residue to adopt a non-canonical conformation with minimal alterations of both the local and global protein structure. Nevertheless, far-UV CD and DSC measurements demonstrate that the Gly52 replacement in TmArgBP^20-233^ has a significant impact on the protein conformational stability. The decrease in denaturation temperature (~8 °C) is comparable to that induced by major manipulations of the protein structure (Fig. 2)^20,21,23^. Indeed, the destabilization of TmArgBP upon truncation of the swapping element, the C-terminal helix, with consequent monomerization of the protein lowers the T_d_ value of 9 °C^21,23^. Moreover, the individual domains generated through a further dissection of the truncated TmArgBP^20-233^ monomer show limited decreases of T_d_ values compared to the parent protein; the ΔT_d_ is about 9 and 3 °C for D1 and D2, respectively^22^.

The use of a highly thermostable protein such as TmArgBP^20-233^ has allowed a direct and detailed visualization of the structural events associated with the Gly52Ala mutation. Barely detectable local structural alterations are responsible for the observed destabilization, highlighting the role of subtle strain in governing the conformational stability of globular proteins. These findings provide a strong experimental support to the idea that apparently minimal geometrical alterations produce significant energetic effects^40–43^.

Finally, we also shown that widely used force fields in molecular dynamics are not able to reproduce the non-canonical local conformation populated by Ala52. This observation suggests that current force fields should be amended and tuned to better reproduce non-canonical states and so improve their predictive performances. In our opinion, this system could represent a relevant benchmark for more sophisticated computational approaches.

## Materials and Methods

### Protein cloning, expression and purification

A truncated form (residues 20–233) of *Thermotoga maritima* Arginine Binding Protein (TmArgBP) was used in these studies (TmArgBP^20-233^). It was generated by deleting the C-terminal swapping helix and part of the hinge peptide of the protein sequence (UniProt code Q9WZ62). Mutant of this short form of the protein were designed by replacing Gly52 with Ala or Val. The genes encoding the Gly52Ala and Gly52Val mutations were obtained by site directed mutagenesis (Quick change mutagenesis kit of Agilent technologies) of the *TmArgBP*^*20-233*^ gene. Both mutants were expressed by using *E. coli* Rosetta (DE3)2 cells following the procedure described in Ruggiero *et al*.^20^. Resource Q fractions were eluted at different concentrations of NaCl. Then, the mutants were loaded on Superdex S75 16/60 pre-equilibrated in a buffer containing Tris-HCl pH 8.0, and NaCl 150 mM. Only in the case of mutant TmArgBP^20-233_G52A^ we were able to obtain both states: the ligand-free and the arginine bound forms.

### Circular dichroism spectroscopy

Circular dichroism (CD) spectra were collected with a Jasco J-810 spectropolarimeter equipped with a Peltier temperature control system (Model PTC-423-S, Jasco Europe, Cremella (LC), Italy). Far-UV measurements were carried out at protein concentration of 0.2 g/l (8 µM) in 10 mM phosphate buffer, at 20 °C temperature, using a 0.1 cm optical path length cell. The range of the reported wavelengths (190–260, 195–260, 200–260, or 205–260 nm) in the far-UV CD spectra was fine-tuned as function of the observed HT voltage. The spectra, recorded with a time constant of 4 s, a 2 nm bandwidth, and a scan rate of 10 nm min^−1^, were signal averaged over at least three scans. The baseline was corrected by subtracting the complete buffer spectrum. The final spectra were expressed as molar ellipticity [θ] (deg cm^2^ dmol^−1^) *per* residue.

The temperature of the transition midpoint (T_d_) was determined by monitoring the change in ellipticity at 222 nm, on increasing the temperature from 20 °C to 100 °C, on protein samples to which 4 M GuHCl was added. The reversibility of the transition was checked by lowering the temperature to 20 °C and re-scanning. The curve was registered using a 0.1 cm path length cell, a protein concentration of 0.2 g/L (8 µM), and a scan rate of 1.0 °C min^−1^.

### Differential scanning calorimetry analysis

DSC measurements were performed on a Nano-DSC (TA Instruments) under a total pressure of 3.0 atm applied with nitrogen gas to both cells in the calorimeter. Protein samples were prepared with an exhaustive dialysis against phosphate buffer saline (PBS) solution at pH 7.4 before each measurement; a scan rate of 1 °C min^−1^ was used for all DSC measurements. Buffer-buffer scans were subtracted from the DSC scans recorded for protein samples. The excess molar heat capacity function (∆C_p_) was obtained after a baseline subtraction, assuming that the baseline is given by the linear temperature dependence of the native-state heat capacity. The denaturation temperature (T_d_) corresponds to the maximum of the DSC peak whereas the denaturation enthalpy change (Δ_d_H(T_d_)) is the integrated area under the DSC peak. The error on the T_d_ values was 0.5 °C and it amounted to 5% in the case of the reported Δ_d_H(T_d_) numbers. The van’t Hoff enthalpy change is calculated by the commonly used formula^28^. The finding that the calorimetric to van’t Hoff enthalpy (Δ_d_H(T_d_)^vH^) ratio, called cooperative unit CU (CU = Δ_d_H(T_d_)/Δ_d_H(T_d_)^vH^), is close to one is a necessary condition to state that the temperature-induced denaturation is a two-state transition^29^. The Nano-Analyze software package supplied by the manufacturer and in-house programs has been used for data analysis.

### Protein crystallization and data collection

Crystallization of the arginine-bound form of TmArgBP^20-233_G52A^ was performed at 293 K by hanging-drop vapor diffusion methods. A screening/optimization of crystallization conditions was achieved using a variety of different conditions. The best crystals were obtained using a protein concentration of 6 mg ml^−1^ in a solution of 2.0 M ammonium sulfate as precipitating agent in a 0.1 M Tris-HCl buffer (pH 8.5).

Diffraction data were collected in-house using a Rigaku Micromax 007 HF generator, equipped with a Saturn944 CCD detector at 100 K. The data were collected by flash-cooling in the supercooled nitrogen gas produced by an Oxford Cryo-system after the addition of 10% glycerol to the harvesting solution. The crystals belonged to the C2 space group and diffracted up to 1.64 Å, although the completeness of the last shell (1.70–1.64 Å) is somehow limited (79.5%). The data set was scaled and merged using the HKL2000 program package^44^. Statistics of data collection are reported in Table 3.

### Structure refinement and validation

The structure of TmArgBP^20-233_G52A^ has been solved by molecular replacement using Phaser^45^ and the structure of the arginine-bound form of the truncated TmArgBP as starting model (PDB ID: 6GGV)^21^. To avoid any structural bias residues 40–55 were removed from the starting model. A preliminary inspection of the electron density clearly indicated the presence of an Ala residue in position 52 (Fig. S11). This region was manually rebuilt in the electron density in the early stages of the refinement. Crystallographic refinement of the structure of TmArgBP^20-233_G52A^ was carried out against 95% of the measured data using the program REFMAC^46^ in the ccp4i program suite. The remaining 5% of the observed data, which was randomly selected, was used in R_free_ calculations to monitor the progress of refinement. Water molecules were incorporated into the structure in several rounds of successive refinement. The final model presents R-factor and R-free values of 0.163 and 0.189, respectively. The basic stereochemistry of the model was checked by using the program Procheck^31^, Molprobity^30^, and an innovative approach based on the monitoring of fine details of the local peptide geometry^40,41,47–51^ (Table S1). In detail, we evaluated the dependence on the conformation of geometrical parameters (valence bond angles and the peptide bond planarity) that are not biased by restraints in the crystallographic refinement (Fig. S12 and Table S3). The variability of these parameters was then compared with that observed in high resolution protein structures by performing a regression analysis (Vitagliano *et al*. in preparation). As shown in Table S3 the variability of NC^α^C, C^β^C^α^C, C^α^CO, C^α^CN_+1_, OCN_+1_, C_−1_NC^α^, and Δω well follows the one detected in high resolution structures.

A summary of the refinement statistics is reported in Table 4. Atomic coordinates of the arginine-bound TmArgBP^20-233_G52A^ protein have been deposited in the PDB with identification code 6Q3U.

### Molecular dynamics

Molecular Dynamics (MD) simulations were performed on the arginine-bound TmArgBP^20-233^ and TmArgBP^20-233_G52A^ mutant using the GROMACS software package 2016.1. AMBER99SB, CHARMM27, and OPLS-AA were used as representative of the most widely used force fields in MD simulations^36^. The model was immersed in triclinic boxes of dimensions 7.14 × 6.98 × 6.67 filled with water molecules (Table S2**)**. The simulations were run for 150 ns with a time step of 2 fs. Six sodium counterions were added to balance charges. The simulations were performed applying periodic boundary conditions. The systems were equilibrated at the temperature of 300 K and the pressure of 1 atm. We first minimized energies by fixing the protein atoms and subsequently without any restraint. The Particle Mesh Ewald (PME) using a grid spacing of 0.16 nm was used to calculate the electrostatic interactions. For Lennard-Jones interactions a cutoff of 10 Å was applied. Bond lengths were constrained using the LINCS algorithm. The analysis of trajectory structures was performed by using GROMACS routines. The achievement of an adequate convergence in the MD simulations was checked by calculating the root mean square inner product (RMSIP) between the two halves of the equilibrated trajectories (50–100 ns and 100–150 ns) considering the motion of C^α^ atoms along the first 10 eigenvectors^52^. A summary of the parameters of the simulations is reported in Table S2. A second independent run of 150 ns was performed for TmArgBP^20-233_G52A^ with all force-fields.

## Supplementary information

Supplementary Material
