# Supplementary material for: The characterization of Thermotoga maritima Arginine Binding Protein variants demonstrates that minimal local strains have an important impact on protein stability
Source: Sci Rep. 2019 Apr 29;9:6617. doi: 10.1038/s41598-019-43157-y (PMC6488590; doi:10.1038/s41598-019-43157-y)
Supplement: Supplementary file 1 — Supplementary Material [file 41598_2019_43157_MOESM1_ESM.pdf]

Supplementary Material

The characterization of *Thermotogamaritima* Arginine Binding Protein variants demonstrates that minimal local strains have an important impact on protein stability

Nicole Balasco[a‡], Giovanni Smaldone[b‡], Marilisa Vigorita[c], Pompea Del Vecchio[d], Giuseppe Graziano[c], Alessia Ruggiero[a*], Luigi Vitagliano[a*]

[a]Institute of Biostructures and Bioimaging, CNR, Via Mezzocannone 16, Napoli, Italy;

[b]IRCCS SDN, Via Emanuele Gianturco 113, Napoli, Italy;

[c]Department of Science and Technology, University of Sannio, via Port'Arsa 11, Benevento, Italy.

[d]Department of Chemical Sciences, University of Naples Federico II, via Cintia, Napoli, Italy.

[‡]These authors contributed equally.
*Corresponding authors. E-mail addresses: luigi.vitagliano@unina.it (L. Vitagliano), alessia.ruggiero@unina.it (A. Ruggiero).

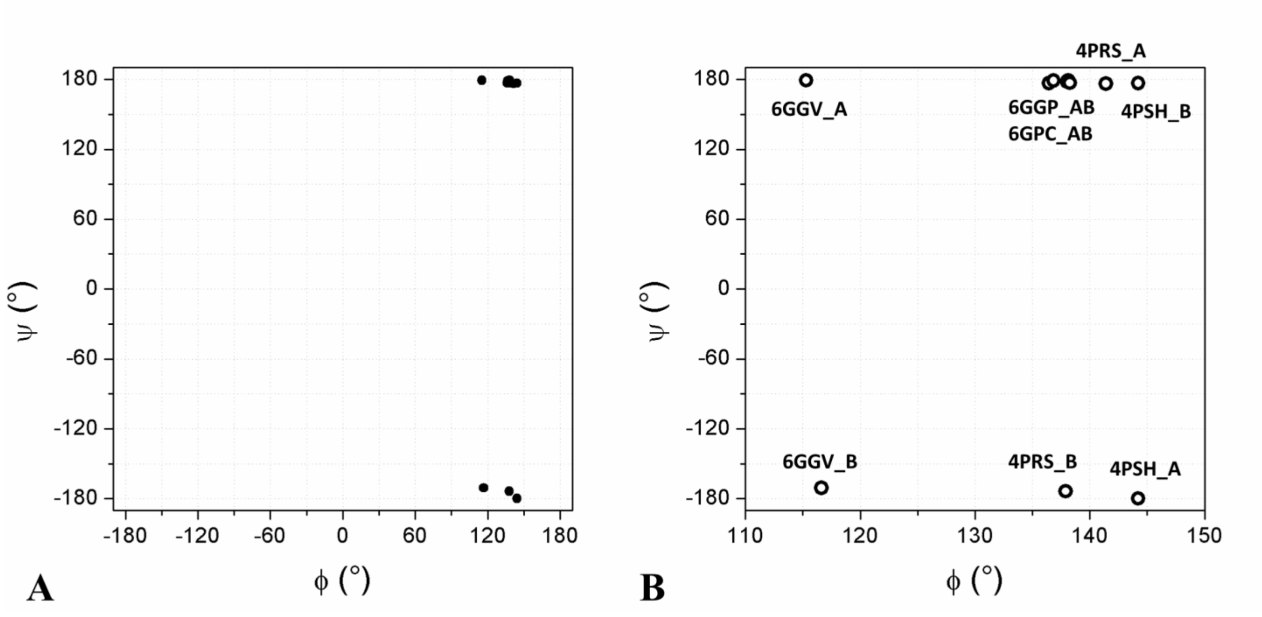

Figure S1. Distribution of Gly conformations detected in TmArgBP structures in the Ramachandran space (A). The PDB codes (and the protein chain) of the different protein forms (dimer, monomer, single D1 domain) are reported in panel B.

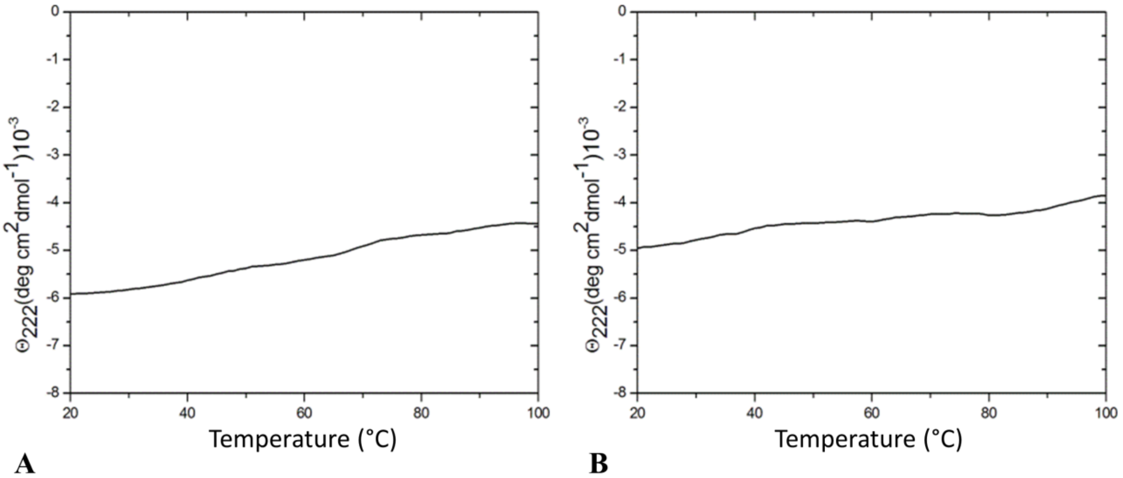

A B

Figure S2. Thermal denaturation profiles of TmArgBP$^{20\text{-}233_G52A}$ (A) and TmArgBP$^{20\text{-}233_G52V}$ (B) in the ligand bound state. The change in ellipticity [θ] at 222 nm when the temperature increases from 20 °C to 100 °C was monitored. The samples were dissolved in PBS buffer at pH 7.4.

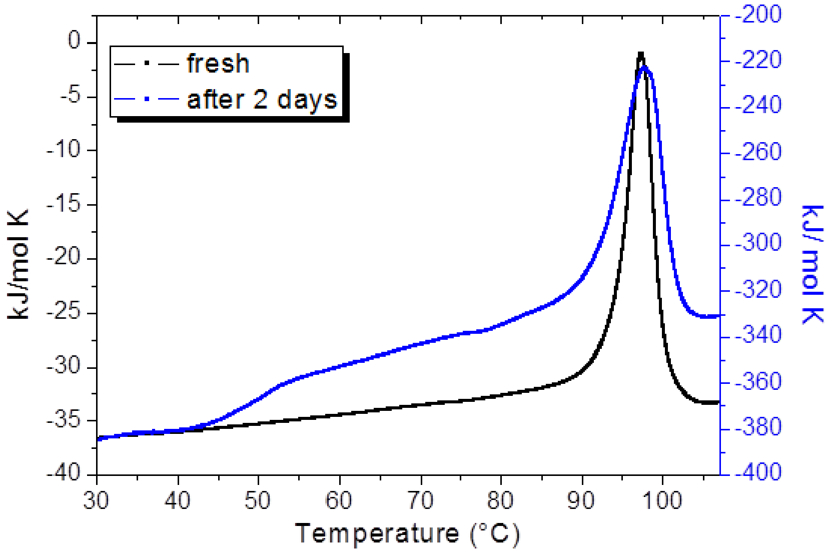

Figure S3. DSC curves of arginine-bound $TmArgBP^{20\text{-}233\_G52V}$ without baseline subtraction. The black line is the DSC profile of the fresh sample whereas the blue curve is the profile of the sample store for two days at 4°C. The samples were dissolved in PBS at pH 7.4.

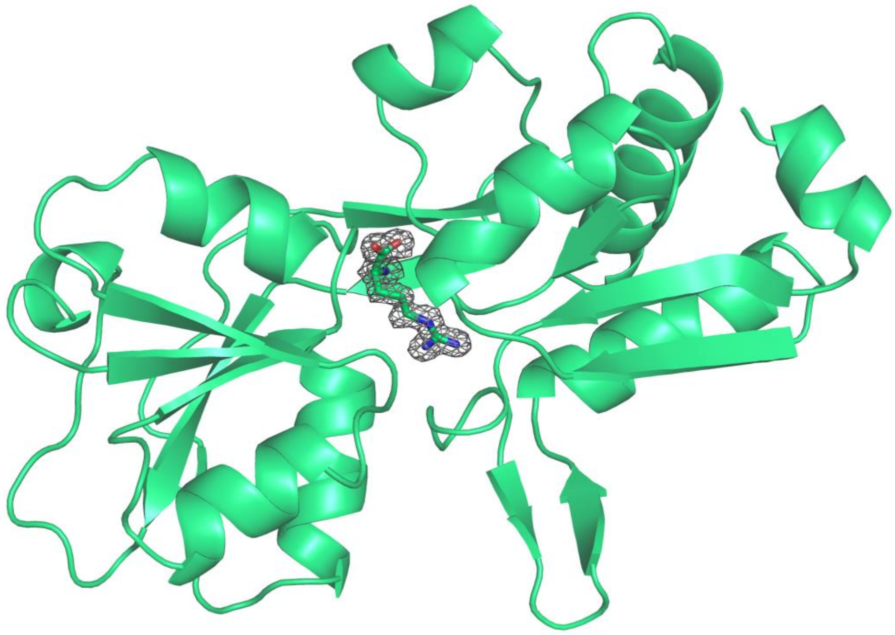

Figure S4. Crystallographic structure of arginine-bound TmArgBP$^{20\text{-}233_G52A}$.The omit |2Fo−Fc| electron density map, contoured at 1σ, of the arginine ligand in the binding pocket is shown.

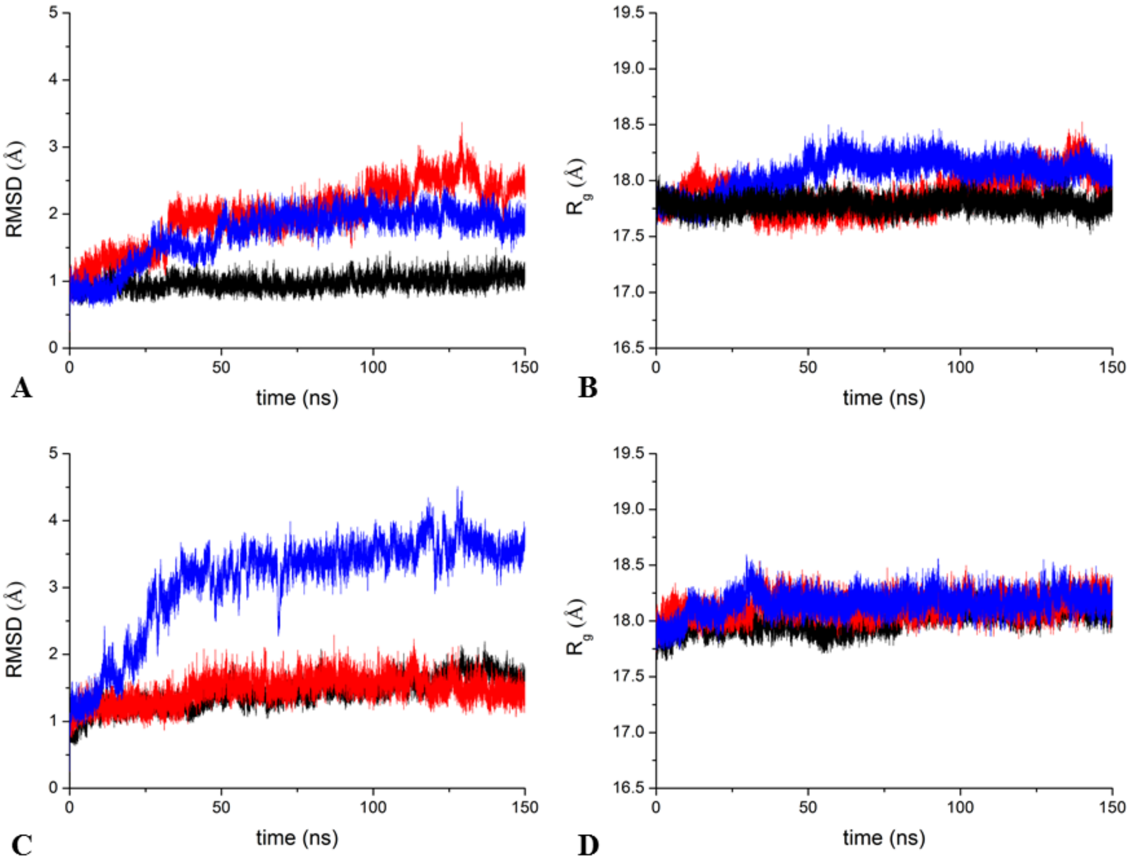

Figure S5. Time evolution of RMSD values and gyration radii of TmArgBP$^{20\text{-}233}$ (A, B) and TmArgBP$^{20\text{-}233_G52A}$ (C, D) in the MD simulations performed with three different force fields: AMBER99SB (black), CHARMM27 (red), and OPLS-AA (blue).

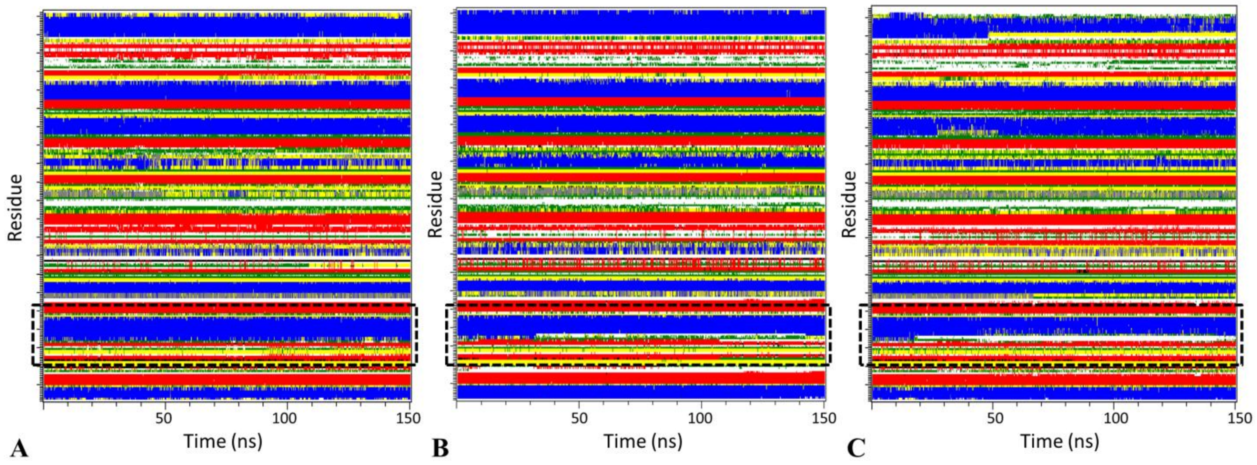

Figure S6. Time evolution of secondary structure elements (α-helices in blue, β-sheets in red, 3-10 helices in grey, turn in yellow, coil in white, bend in green, β-bridge in black) in the MD simulations of TmArgBP$^{20\text{-}233}$ performed with three different force fields: AMBER99SB (A), CHARMM27 (B), and OPLS-AA (C). The region of the helix insertion motif (residues 40-70) is indicated by a black box.

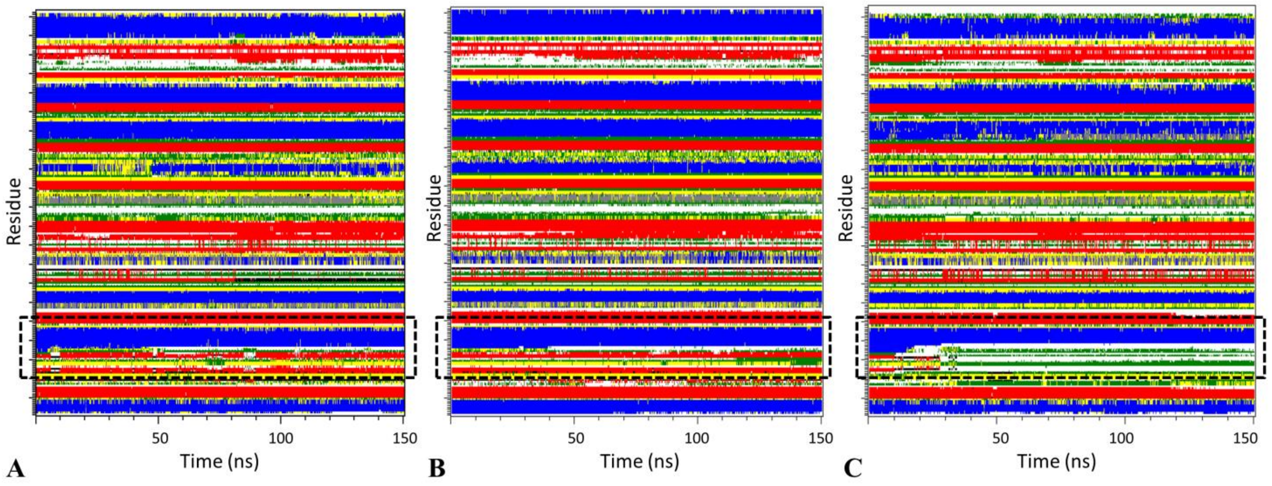

Figure S7. Time evolution of secondary structure elements (α-helices in blue, β-sheets in red, 3-10 helices in grey, turn in yellow, coil in white, bend in green, β-bridge in black) in the MD simulations of TmArgBP$^{20\text{-}233_G52A}$ performed with three different force fields: AMBER99SB (A), CHARMM27 (B), and OPLS-AA (C). The region of the helix insertion motif (residues 40-70) is indicated by a black box.

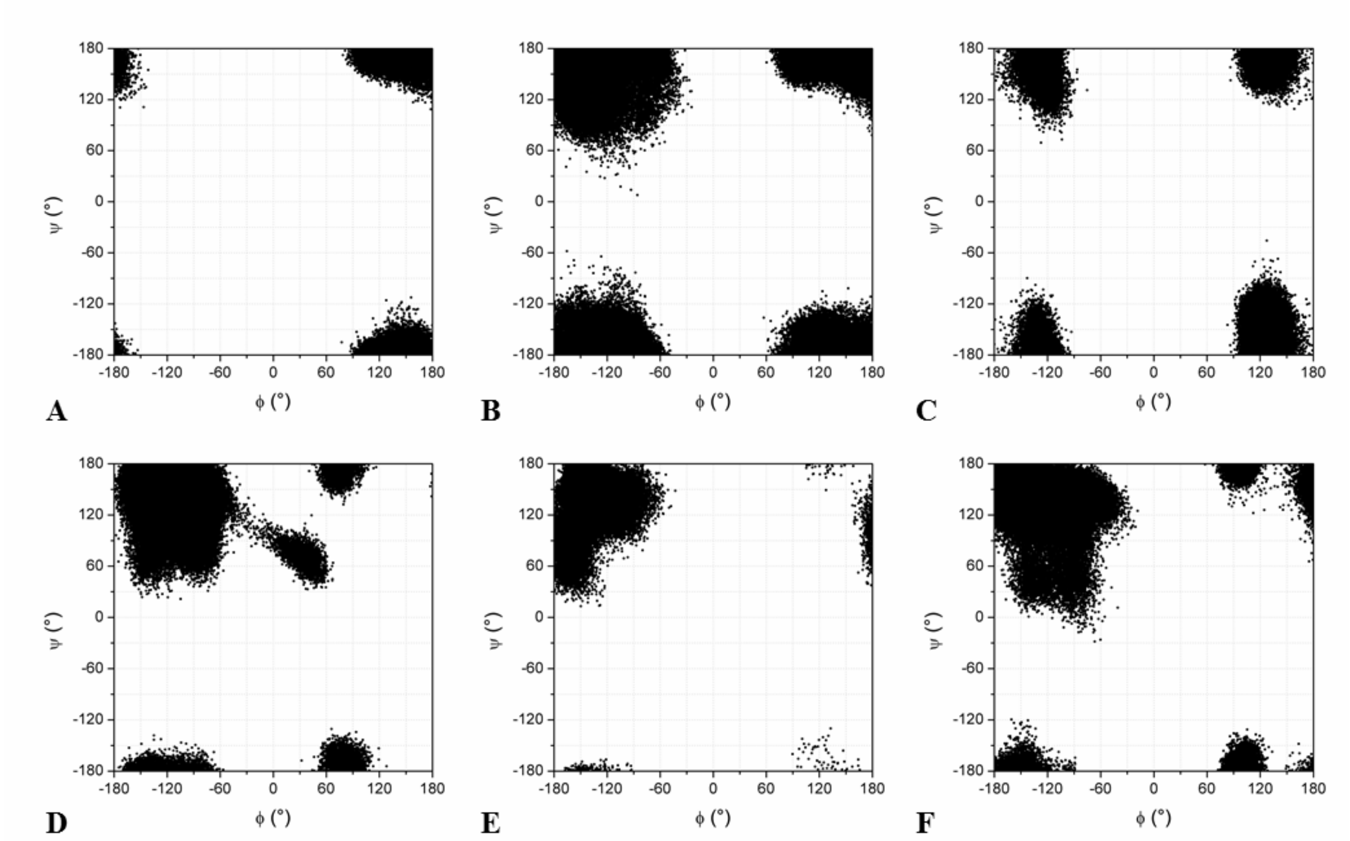

Figure S8. Ramachandran plots showing the conformations adopted by Gly52 (A-C) or Ala52 (D-F) in the trajectory structures in the simulations of TmArgBP$^{20\text{-}233}$ and TmArgBP$^{20\text{-}233_G52A}$ performed with three different force fields: AMBER99SB (A, D), CHARMM27 (B, E), OPLS-AA (C, F).

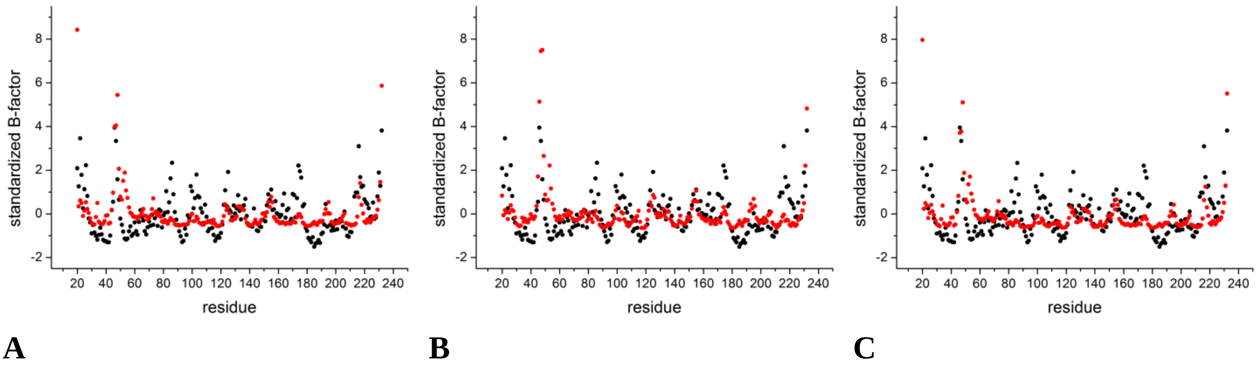

Figure S9. Pairwise comparison of standardized experimental B-factors of TmArgBP$^{20\text{-}233_G52A}$ crystal structure (black) with those computed in the MD simulations of TmArgBP$^{20\text{-}233_G52A}$ (red) performed with three different force fields: AMBER99SB (A), CHARMM27 (B), and OPLS-AA (C). Standardized B-factors (BN) are calculated for the protein C^{α} atoms by using the following definition: $BN=(B-B_{av})/B_s$ where B_{av} is the average B-factor and B_s is its standard deviation.

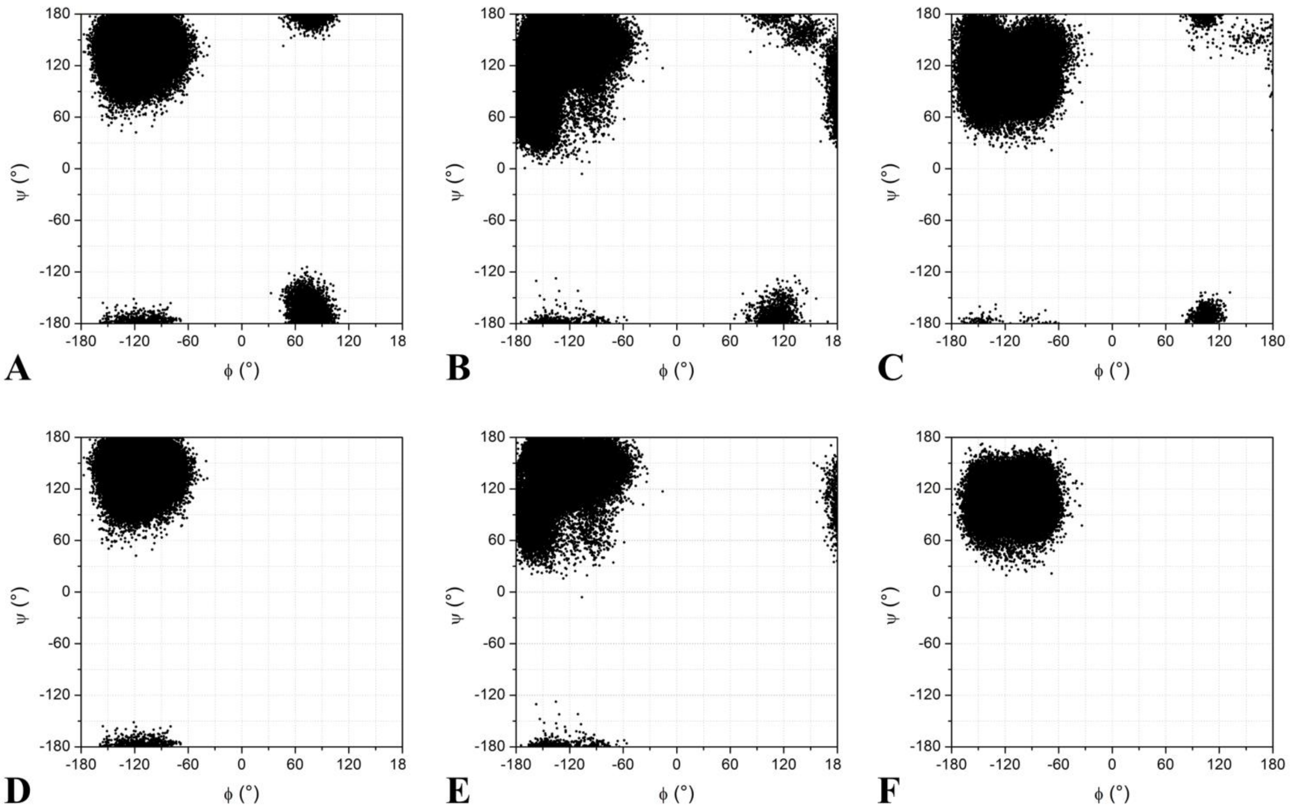

Figure S10. Ramachandran plots showing the conformations of TmArgBP$^{20\text{-}233_G52A}$ Ala52 in all trajectory structures of the second MD run performed with AMBER99SB (A), CHARMM27 (B), OPLS-AA (C) force fields and in trajectory structures obtained in the equilibrated region of trajectories (50-150 ns) obtained with AMBER99SB (D), CHARMM27 (E), OPLS-AA (F).

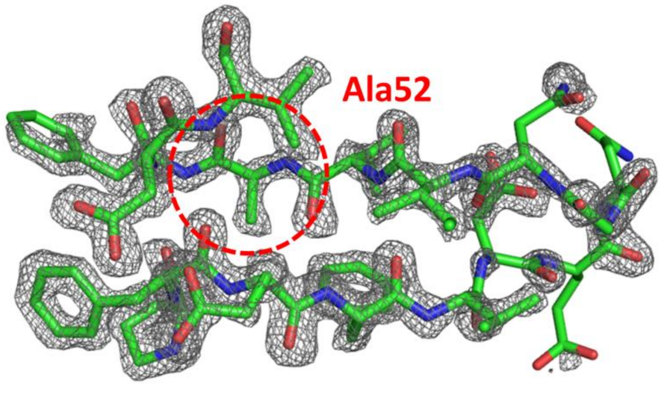

Figure S11. |Fo-Fc| omit map, contoured at 3σ, of the region carrying the mutation Gly52Ala (residues 40-55) in the crystal structure of the arginine-bound form of $TmArgBP^{20\text{-}233\_G52A}$ obtained just after the molecular replacement step.

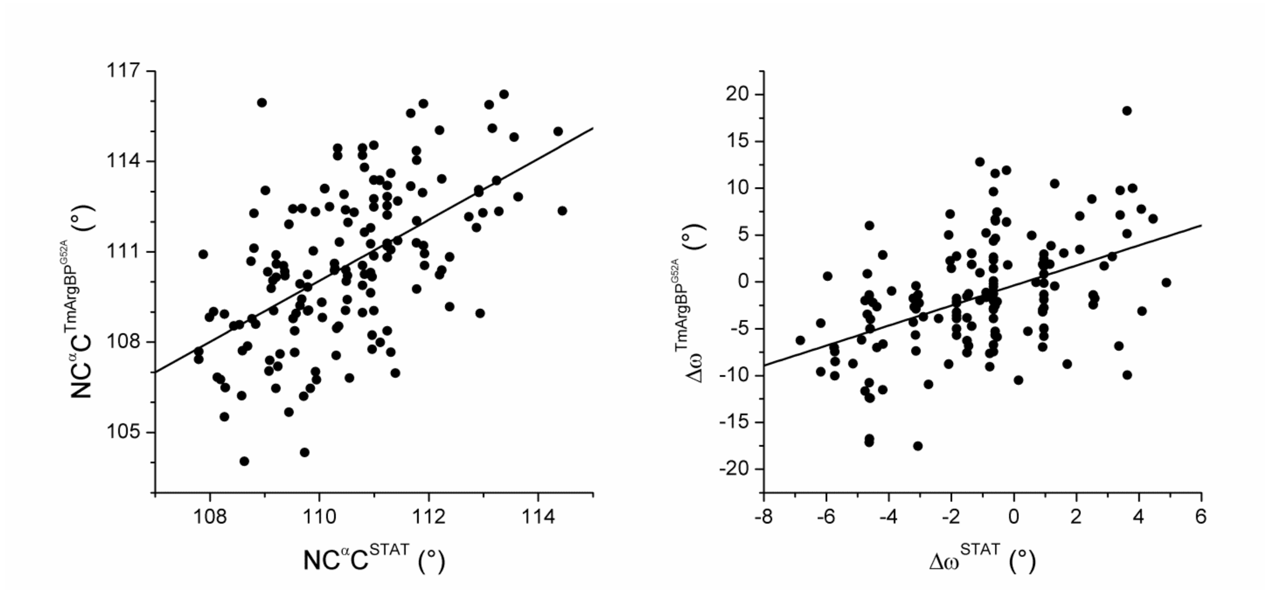

A **B**

Figure S12. Validation of the variability of some geometrical parameters of the protein backbone: the NC$^{\alpha}$C bond angle (A) and the deviation from the peptide bond planarity $\Delta\omega$ defined as (ω-180°)mod360° (B) of TmArgBP$^{20\text{-}233_G52A}$ crystal structure. The regression analysis has been performed by plotting the NC$^{\alpha}$C or $\Delta\omega$ values of TmArgBP$^{20\text{-}233_G52A}$ residues *versus*the average NC$^{\alpha}$C or $\Delta\omega$ values of residues adopting the same (φ, ψ) conformation obtained from databases of PDB X-ray protein structures solved at high resolution (release of March 2016). These structures were selected using the PISCES culling server (http://dunbrack.fccc.edu/PISCES.php) applying specific criteria: resolution better than 1.6 Å for bond angles or 1.2 Å for dihedral angles, R-factor ≤ 0.20, and sequence identity ≤ 25%. Additional selections were carried out at residue level by excluding residues for which the ratio between the average backbone B-factor (atomic displacement parameter) of the residue and the same parameter computed considering the entire chain was > 1.3. These databases contain 3,291 (database of bond angles) and 799 (database of dihedral angles) non-redundant protein chains. The regression lines are shown. The parameters (correlation coefficient and p-value) of the linear fitting are reported in Table S3.

Table S1. Local geometry of Ala52 residue in the crystal structure of TmArgBP$^{20\text{-}233_G52A}$. For comparison the average values obtained from databases of X-ray protein structures solved at high resolution (resolution <1.6 Å for bond angles and resolution < 1.2 Å for $\Delta\omega=(\omega - 180°) \bmod 360°$) are also reported.

Ala52 angles (°)		Statistical Average Values and Standard deviations (°)
**φ**	117.3	-
**ψ**	-170.9	-
**ω**	-162.8	-
**ω_{-1}**	162.9	-
**Δω**	17.2	-0.99 ± 11.2
**$NC^{\alpha}C$**	110.6	110.8 ± 2.4
**$NC^{\alpha}C^{\beta}$**	116.3	110.7 ± 1.4
**$C^{\beta}C^{\alpha}C$**	113.4	110.2 ± 1.7
**$C^{\alpha}CO$**	118.3	120.5 ± 1.0
**$C^{\alpha}CN^{+1}$**	117.4	116.7 ± 1.3
**OCN^{+1}**	123.5	122.8 ± 1.0
**$C^{-1}NC^{\alpha}$**	127.4	121.4 ± 2.0

Table S2. Parameters and statistics of MD simulations performed on TmArgBP$^{20\text{-}233}$ andTmArgBP$^{20\text{-}233_G52A}$.

System	Force field/water model	Box dimensions (nm^3)	No. of water molecules	RMSIP[a]
TmArgBP$^{20\text{-}233}$	AMBER99SB/tip4pew	7.14x6.98x6.67	9777	0.79
	CHARMM27/tip3p		9976	0.76
	OPLS-AA/tip4p		9977	0.84
TmArgBP$^{20\text{-}233_G52A}$	AMBER99SB/tip4pew		9775	0.78
	CHARMM27/tip3p		9977	0.86
	OPLS-AA/tip4p		9975	0.84

[a]The RMSIP values have been calculated by dividing the last 100 ns of each trajectory in two equivalent halves.

Table S3. Results of the regression analysis of the geometrical parameters of TmArgBP$^{20\text{-}233_G52A}$ compared to those found in high resolution crystal structures (resolution <1.6 Å for bond angles and < 1.2 Å for dihedrals) (see legend of Figure S12). The peptide bond deviations from planarity $\Delta\omega$ is defined as $(\omega - 180°) \bmod 360°$ whereas the carbonyl carbon pyramidalization θ_C is defined as $(\omega - \omega_3 + 180°) \bmod 360°$ (ω_3 is the dihedral angle defined by the atoms $OCN_{+1}C^{\alpha}_{+1}$).

angles (°)	Correlation coefficient R	p-value
**ω**	0.47	$1.1*10^{-9}$
**θ_C**	0.13	0.12
**$NC^{\alpha}C$**	0.57	$1.8*10^{-14}$
**$NC^{\alpha}C^{\beta}$**	0.14	0.08
**$C^{\beta}C^{\alpha}C$**	0.36	$4.7*10^{-6}$
**$C^{\alpha}CO$**	0.24	$2.5*10^{-3}$
**$C^{\alpha}CN^{+1}$**	0.39	$5.1*10^{-7}$
**OCN^{+1}**	0.26	$1.1*10^{-3}$
**$C^{-1}NC^{\alpha}$**	0.41	$2.0*10^{-7}$
